# Address to the Graduating Class of the Baltimore College of Dental Surgery

**Published:** 1847-06

**Authors:** Eleazar Parmly


THE AMERICAN

### Journal of Dental Science.

Vol. VII.] JUNE, 1847. [No. 4.

### ARTICLE I.

### *Correspondence.*

Baltimore, *January 12th,* 1847.

My Dear Doctor,

I am requested by the Faculty of the Baltimore College of Dental Surgery, to inquire if it would be convenient for you to be present at the next Annual Commencement of said Institution; and if so, if you would act as Chairman of a Committee to award a case of instruments to the graduating student who shall exhibit the highest proficiency. We are exceedingly anxious that you should be present on the occasion, and participate in the ceremonies. The commencement will be held on the 15th and 16th of February. Yours, most truly and affectionately,

(Signed,) C. A. HARRIS.

To Dr. E. Parmly, *N. Y.*

New York, *February 9th,* 1847.

Dear Sir:

In my last letter I did not accept, formally, the very flattering invitation from the Faculty to be present at the examination of the graduating class, for I was afraid that something might happen to prevent my fulfilling the duty assigned me on the Committee appointed to bestow the premium due to industry and professional attainments.

Dr. Roper has promised to accompany me, and we shall most likely be in Baltimore on Saturday ensuing.

Please offer my most grateful thanks to the Faculty, for this distinguished mark of their respect and good will; and, with the highest consideration for the talents and virtues of your associates in the institution, I am,

Sincerely and affectionately, your friend,

(Signed,) ELEAZAR PARMLY.

TO DR. C. A. HARRIS.

---

DENTAL COLLEGE, BALTIMORE,
*February, 17th,* 1847.

DEAR SIR:

The graduated class of Dental Surgeons of the Baltimore College feel anxious that you would be pleased to allow your valuable address in full, as delivered by you in the College, on Tuesday evening, the 16th inst., to be published in connection with the addresses of Dr. Westcott and Dr. Bond, and that a copy of the same be furnished to the editor and directors of the Journal of Dental Science, at your earliest convenience.

Dear sir, we remain,

Yours, very respectfully,

(Signed,) JAMES H. McCULLOH,
JOHN D. WEMPLE,
JOHN N. BAIRD,
*Committee.*

TO DR. E. PARMLY.

---

BARNUM'S HOTEL, BALT., *Feb'y 17th,* 1847.

GENTLEMEN:

Few circumstances have occurred, since I entered upon the duties of our profession, that have gratified me more than observing the various objects and advantages connected with the institution from which you have just received your diplomas—the reward of your industry, and the proof of your attainments in professional art and science. The address which you have, in so complimentary a manner, requested of me for publication, is entirely at your service. It was intended expressly for the class, and not for a mixed assembly or for publication, as I said to the audience last evening; but written in the hope that, by enumerating some of my early struggles, they might be to you incentives to press on and overcome difficulties. Years of experience, and a heavy expenditure of money to obtain a professional education, did not give me the advantages, in early life, that you have enjoyed in acquiring knowledge, and in applying that knowledge by manual dexterity, to the relief of suffering, and to remedying and supplying defects occasioned by accident, malformation or disease.

With high admiration for the cleverness which manifested itself upon your examination, and with every kind wish for your future prosperity, I am, gentlemen, Very truly and respectfully yours,

(Signed,) ELEAZAR PARMLY.

To Messrs. McCulloh, Wemple and Baird, *Committee.*

---

Baltimore, *March 5th,* 1847.

My Dear Doctor:

As the official organ of the Baltimore College of Dental Surgery, it is my duty to inform you that the Faculty are especially desirous of having your address for publication, together with the report of the awarding committee, of which you were the chairman.

It gives me pleasure to be the medium of communicating the thanks of the Faculty to you, and through you to the awarding committee, for the patient, faithful and impartial manner in which the committee discharged the arduous duties which they had the kindness to undertake.

Yours, very respectfully,

(Signed,) W. R. HANDY.

To E. Parmly, M. D.

---

New York, *March 10th,* 1847.

My Dear Sir:

The highly complimentary letter which I have had the honor of receiving from you, in behalf of the Faculty of the Baltimore College of Dental Surgery, requesting permission to publish the short address delivered before the graduates of that Institution, at its late commencement, is gratifying to my feelings, and particularly so as it contains an assurance that my desire to serve the Institution, in conjunction with an equal ardor on the part of my worthy and highly esteemed colleagues, Drs. Roper and Noyes, was kindly regarded by you.

I promised the graduates, on the morning after commencement, in answer to a very kind and respectful note received from them, that I would with pleasure furnish them a copy. Although there is much in it that would afford no interest to any but personal friends, yet, if any portion of it shall excite them to industry and perseverance, I shall be amply gratified, and my aim in writing it will be fulfilled.

With the highest respect for the Faculty, and with warmest wishes for the entire success of your praiseworthy efforts in your noble undertaking,

I am, sincerely and respectfully, yours,

(Signed,) ELEAZAR PARMLY.

To Dr. W. R. Handy, *Baltimore.*

*Address to the Graduating Class of the Baltimore College of Dental Surgery, at its Seventh Annual Commencement*, February, 1847.

My youthful friends, with honors graced,
The meed of industry and taste—
The just reward of toil severe
Through many a long and busy year—
The promise fair of noble aims,
Such as the social welfare claims—
Indulge my Muse in humble song,
Which—good or bad—will not be long;
A virtue few are prone to teach,
Of those who sing, or those who preach.
I write, as some may well suppose,
In careless verse instead of prose,
Because they know I've not the time,
To write in any thing but rhyme;
Thus, should you find my rhyming tame,
The muse, not I, must bear the blame.

As earth's brief years are rolling fast,
And soon with me will all be past—
My hours of earthly toil be done,
My labors cease, my race be run—
To arm you for the manly strife,
I'll read the story of my life.
No proud descent from rank I claim,
No princely wealth—no warrior fame
Exalts the names, or gilds the page,
From which I trace my lineage.
Yet there are those whose virtues shine
As bright, along the ancestral line,
As e'er a monarch's of the earth;
In admiration of whose worth,
I would in feeble words impart
The tribute of a grateful heart.

WHEELOCK! revered and honored name!
The source from which my being came,
To thy blest memory I owe
All that affection can bestow,
And here I give my meed of praise
To him* whose virtues strove to raise,
Instruct, improve, emancipate
The Indian from his abject state,
Who justly owned his injured cause,
And fenced him with protective laws:
Who, by his worth and fair renown,
A charter gained from England's crown,
To rear a college, and impart
The light of science and of art
To nature's sons—a noble band,
The rightful owners of the land—
To aid them in their manly toil,
To clear the land and till the soil;
To teach their minds to soar on high
To nature's God, who rules the sky,
Who rolls His thunder round the world,
'Mid lightning-banners wide unfurled!
Such—and thus worthy to be sung—
The noble men from whom have sprung
New England's hardy yeomanry,
Enlightened, virtuous, brave and free—
Forth borne on emigration's tide
To people regions far and wide—
Well known where'er their cabins stand
As pilgrims from the father-land.

* ELEAZAR WHEELOCK, S. T. D.
Founder and First President of Dartmouth College,
and Moor's Charity School.
By the Gospel he subdued the ferocity of the Savage—and to the Civilized he opened new paths of Science.
TRAVELLER,
Go, if you can, and deserve the sublime reward of such merit.
*Vide Wheelock's Life*, p. 80.

On Monday morn, the thirteenth day
Of March—as family records say—
In seventeen hundred, ninety-seven,
The light of life to me was given,
In Vermont state—in Braintree town;
A spot retired—of no renown—
A wild uncultivated place,
Where panthers fierce pursued their chase,
Through scanty fields and pastures rude
To swamps of darkest solitude,
To mountain high, or forest wood,
And lapped their victim's flowing blood.
The mountains hemmed our çottage round,
Where gloomy forests darkly frowned;
The country all around was new,
With scarce a cot to mark the view,
And there my early years flew by
Without a sorrow or a sigh.
All that I knew of earth's wide span
My boyish vision there could scan;
I knew no other place than home,
Nor felt one wayward wish to roam.

My parents, ever good and kind,
Were both to quiet life inclined.
In steady toil their years were spent,
Yet were they cheerful and content;
No jarring discord e'er was heard,
No angry look, or angry word,
But acts of fond affection seen,
Where all was peaceful and serene.

Four much loved brothers were my lot—
Four sisters shared our lowly cot;
Two elder brothers loved me there,
Two sisters kind my seniors were;
Two junior brothers I enrol,
Two younger sisters make the whole.

In true affection these were mine—
And thus, as children, we were nine.
On those blest years I fondly dwell.
No care had made my bosom swell;
All that I knew on earth were dear,
And all I loved were ever near.
Beyond our home we knew no charm—
The world was then our little farm.
With what keen pleasure I review
The scenes my early childhood knew:
The grassy fields o'er which I strayed,
The pebbly brook in which I played;
My tiny dam, with slips of deal
For water-spout and water-wheel;
Our cottage with its naked floor,
The trees that stood around the door,
The logs, piled up in rudest form
To screen our cattle from the storm,
Which, although few, yet fully tried,
Our wants and wishes well supplied.
The bleating lambs and fleecy flock—
The pasture ground of hill and rock,
The icy spring and meadow rill—
The church that stood upon the hill,
The school-house that was on the way—
The road where children used to play—
The woodland dark, and rushy fen,
The pathway up the narrow glen,
The bridge across the deep ravine
Where piles of mossy rocks were seen,
The sunny hill, the fertile plain,
The little fields of waving grain.
The cottages we had to pass,
With paper-lights instead of glass,
With mudded walls and chambers dark,
With rough-hewn floors and roofs of bark—
Such were the homes I used to see,
And they seemed happy homes to me.

These were the scenes my childhood knew,
Amidst *them* I to boyhood grew.
Deep on my heart their stamp is set,
And memory lingers round them yet.

Before ten happy years had fled,
By ever changeful fortune led,
I left the spot where childhood's hours
Had passed in fields and woodland bowers,
Where peace and plenty had been found,
And joy and comfort spread around,
Where all were happy—all were blessed—
This now by others was possessed.
Thence, wandering sixty miles or more
Across the mountains, near the shore
Of Lake Champlain, we found a land
Of rugged rocks and barren sand—
Of naked hills and stony vales,
Of bushy swamps and marshy swales—
Of frightful cliffs, and caverns deep,
Where foaming torrents hoarsely sweep
O'er beds of rock, deep, overgrown—
Where star or sunlight never shone.
For wilder scenes or rougher ground
Can scarcely any where be found,
Where man has tried, by hardy toil,
To clear the land and till the soil,
Than where my boyhood's lot was cast,
And youth's first years in labor passed.

Till summers twelve had winged their way,
My hours were spent in work and play,
For schools were few and teachers rare,
And scant the means we had to spare;
Terms of three months—"wages and board"
Were all the neighbors could afford—
These terms—at intervals delayed
So long, the little progress made

At one, would, ere the next came on,
From mind and memory be gone,
So with this aid, in all our need,
We barely then were taught to read.

A change brought round a bright event—
To school at Montreal I went,
And greater kindness ne'er was shown
Than I received from one well known;
Of manly virtues, generous worth,
A kinder dwelt not on the earth;*
His heart was bounteous, open, free;
His consort good and kind as he;
A sterling pair of virtuous minds,
Which one on earth so rarely finds;
Who made me feel, though forced to roam
From all I loved of friends and home,
That they had now become to me
All that the best of friends could be.
Two years their friendship thus I shared,
When war with England was declared,
And all who loved their native land,
And by its banner meant to stand,
Howe'er their business might incline,
Were forced to cross the British line—
Or England's royal sceptre own,
And swear allegiance to her throne.
By this, the circle dearly loved
Was broken up, and friends removed,
To join the standard of the brave—
The stars and stripes, that proudly wave
O'er freedom's land by valor won—
The gift of Heaven, through Washington!
Who first the flag of Britain furled,
Proclaiming boldly to the world,
That here we spurn a tyrant's nod,
And own no monarch but our God!

* Major Levi Mower.

I still remained, too young for both,
Not taking either arms or oath ;
My ardent wishes thus destroyed—
The passing time I next employed
Where art and learning sweetly blend,
Within the office of a friend—*
Who was, in many a worthy deed,
The friend well proved in time of need ;
And every feeling of my heart
Was bent to learn his noble art.

But soon another change was made,
My parents now required my aid,
And sent me word to come and try,
With them, by labor to supply
The wants of those that round them clung—
Brothers and sisters—dear and young.
With gladdened heart the wish I heard,
From those whom truth and love endeared ;
And soon the scene I left behind,
Where ardor still had urged my mind
To the fond hope that I might rise,
Through this prospective enterprise—
Above the hard and humble sphere
Of toiling on from year to year—
Repaid but by the scanty spoil
Resulting from a rugged soil.

And now the summons to obey,
With all I had I took my way
On foot, and reached the happy place,
And saw once more my father's face.
That meeting in my heart is set—
Its joys I never can forget!
To be with those I loved again,
Repaid me for whole years of pain.

* Nahum Mower, Esq., Editor and Publisher of the Canadian Courant.

To see affection's flowing tears,
From her who watched my early years—
To hear her speak, and see her smile,
And gaze upon me all the while,
As on one risen from the dead—
Her hand still resting on my head,
With all the warmth and tender zeal,
Which—save a mother—none can feel.
All—all these memories, thrilling dear,
Demand the tribute of a tear.

At home awhile, in toil severe,
In cold and wet, in dry and clear,
In scorching sun, 'mid burning sand,
I daily wrought upon the land ;
And whatsoe'er before me lay,
I followed closely, night and day,
Delighted thus to see supplied
The daily wants that man betide.

Another change new duties brought—
A district school was to be taught;
And neighbors met, as was the rule,
To choose a teacher for the school—
And, none dissenting, all agree
That duty to assign to me.
No greener spot on life's broad waste,
Where I by fortune have been placed,
E'en to my memory now appears,
Brighter along the path of years,
Through which from early life I've strayed,
And, step by step, advances made—
Than that on which it oft now dwells,
The place where virtue aye excels,
And gains the rich award of praise—
Sure earnest of its future days—
The school, where kindness does secure
The love of hearts—young, warm and pure.

No happier group was ever seen
Than came to me at seventeen;
Tho' many far my seniors were,
All strove to do their duty there—
And when our winter's term did end,
In each loved pupil, I'd a friend.
This scene remembrance still endears—
For every eye was dimmed with tears,
As sped the hour when we must part,
And grief and sorrow filled each heart.

War's desolation now was o'er,
And smiling peace returned once more;
Our intercourse, so long at stand,
Was opened free by sea and land,
And merry hearts and friendly signs
Again lit up our frontier lines.

With slender means—oppressed with care
Such as the lowly have to bear—
Dependant wholly on the soil,
For bread wrung out by hardest toil,
No brighter hope to cheer life's way
Than labor's poor avails each day—
It now became us to decide,
And for a future home provide.
So, feeling free the choice to take,
Where he his resting place might make,
My father, jointly with the rest,
Decided on the growing West;
And in Ohio's fertile vales,
Where nature's richest soil prevails,
And deep within her forest shade—
On Erie's shore his purchase made.

Meanwhile an elder brother came*
From Boston, where he'd gained the fame

* Levi Spear Parmly, of New Orleans.

Of cleverness in every part,
Pertaining to the Dental Art;
And finding me without employ,
A busy, restless country boy—
Proposed that I should leave the land
And try my then unpractised hand
To imitate, in skill, his own,
By carving teeth from sea-horse bone.

My brother! one might search around,
Thy equal rarely would be found.
A mind with energy replete,
Where all the warm affections meet
And strive with an ambitious will—
The ever-ruling feature still.
Within thy breast, in early day,
A Buonapartean spirit lay;
Th' ambition that in one arose
To rule the nations—curb his foes—
Oft have I thought fell far beneath
Thy mind's devotion to the teeth.

With open hand and liberal heart,
Thou well hast done a brother's part;
And mayest, in truth and justice, claim
From all who bear the common name,
Their gratitude for timely aid,
And bounty stinted not nor stayed.
In many a dark and trying scene,
Our benefactor thou hast been—
As sunbeams, darting from the sky,
The early dews of morning dry,
So thou, my brother! fond and dear,
Hast often dried affliction's tear.

The books then written, that I knew*
On the profession, were but few;

* We have had but few writers in this country even to this day. The American Journal and Library of Dental Science, embodies all that has been published of any

But, each and all, I read with care,
And found this wholesome counsel there,
That great perfection could be gained,
And wealth and honor thus obtained—
If one strict course should be pursued
Of industry and rectitude.

I soon began to take a part,
With trembling hand and throbbing heart;
And giving less, more pain to save—
I often felt more than I gave—
Then from my patients oft would go,
To stifle fears I dare not show.
But time rolled on, and I could stand
By sufferers with a steady hand.

Although my fears were thus subdued,
My practice yet was rough and rude—
And soon I learned from those who came,
Whose works had gained an honored name—
What then was done in other lands,
By men with well-instructed hands,
And to this end my mind was brought
That hands as well as heads are taught—
So to obtain the skill discerned
With strong desire my bosom burned.

For two years more I labored through
A practice such as I could do, real value to the dental literature of this country. The Journal of which your professor Dr. Harris has been the principal editor, taken in connection with his other writings, will make him the largest, as well as the ablest contributor that has yet appeared in the field of American authors.

Dentologia, a poetical essay by Solyman Brown, is a gem by itself, and can hardly be brought into comparison with other works on the profession. It is decidedly the neatest and prettiest essay that has ever been published on the teeth, with which I am acquainted, and the author and the essay well deserve what a distinguished writer has said: "It is written at such a height upon the rocks of poetical truth as to have placed it in all probability beyond the reach of cotemporaneous rivalry."

Where hospitality abounds
Among the growing western towns—
From Pittsburgh on to New Orleans—
Till I had gained sufficient means
To warrant me a voyage to make
To Europe—solely for the sake
Of study there, with men of name,
Who had for skill the highest fame.

'Twas thus with what I had in hand,
I left my loved, my native land;
Resolved with hard-earned gold to part,
To gain perfection in my art.

And now what grateful thoughts arise,
When bright-winged memory backward flies;
And brings around me scenes of youth,
Investing them with life and truth!
And no where have I ever felt
Friendship more dear than when I dwelt
Within the sphere, where Clay has won
His brightest honors—Lexington.

While freedom's banner waves unfurled,
His name will echo round the world—
Marking the region of his birth
The fairest spot on all the earth.
And Ashland, too—the statesman's home—
A pilgrim shrine in years to come,
Shall long transmit the honored name
And share the splendor of its fame.

Fair land of Boone! I love thee still,
Thy grassy vale—thy oak crowned hill—
Thy balmy air—thy verdant plains
Where universal plenty reigns—
Thy lofty mounds o'er warriors' graves,
Thy forests dark—thy vaulted caves—

Still move o'er memory's magic glass,
And bring fresh transports as they pass.
The welcome word, the friendly hand,
Heard and received in all the land—
The warmth with which thy children greet
The lonely stranger whom they meet—
Their grief unfeigned, when friends depart
Have stamped thy worth upon my heart!

Our art was there unknown to fame,
Save such as served to mar its name,
Yet tho' in rank degraded low,
Warm hearts and hands did there bestow—
Above the pride of wealth and strife—
The courtesies of social life.

I oft had heard, in earlier days,
Of hospitality—the phrase
Was oft familiarly set forth
By our good people of the north,
In all the studied forms of art
That show the head and not the heart—
Yet, till to southern climes I went,
I knew not what the language meant.

Where'er I rove—where'er I rest,
While feeling dwells within my breast,
And thought and reason still retain
Their wonted empire in the brain—
In gratitude for good to me,
Kentucky! I'll remember thee!

With many a kind and grateful thought
To those, who thus their aid had brought
For services of little worth,
Compared with what are now sent forth;
And finding here no one to teach
That skill which seemed beyond my reach,

That no fair fee could then command
Instruction from an able hand;
With no such teacher in my view—
I then resolved to bid adieu
To home, to friends and native skies,
And seek—what all good dentists prize—
True knowledge—by which art is made
Conducive still to human aid.

The ocean now before me lay,
And o'er it seemed the only way;
Hope sprang unto the other side,
And whispered she would there provide
The opportunities desired
To gain the knowledge I required.
Then o'er the Atlantic's bounding tide
Did the good vessel Marmion glide,
And bore me to the white-cliffed coast—
A noble nation's pride and boast.
Straight to the field of virtues rare,
To London—I did then repair;
A scene of boundless wealth and show,
And of the direst depths of woe!
Of royal parks and princely squares,
Of alleys, courts and thoroughfares;
Of gorgeous temples and hotels,
Of beggars' hovels—gamblers' hells;
Of all that's high in rank or worth,
And all that's low in mind and birth:
The seat of all that's grand in style,
And all that's mean, debased and vile.
These wide extremes all strangers greet,
And more than these in London meet.
Hither ambition led my way,
And here I witnessed, day by day,
The works of men in standing high,
On whose fair fame I could rely;

Who were in practice then best known,
Men of prime worth, and manly tone;*
In art and science who maintained
The rank their sterling merits gained;
Till satisfied whilst there I'd been,
I next resolved to change the scene.

To Paris, then, my steps did turn
The modes of practice there to learn;
And find if there were greater names
Than those I honored on the Thames.
With conscious pleasure I recall
The noblest mind amongst them all,
Of those most willing to impart
A knowledge of their useful art:
And whilst I live, will justice claim
My gratitude to Maury's name.†

At length my time with Maury o'er,
To London I returned once more,
And urged by friends to memory dear—
With feelings warm and hearts sincere—
Their confidence and gifts to share,
I, for a time, resided there,

* The gold stoppings of Waite were decidedly the best of any I saw in England, and I have never seen any since that surpassed them in beauty of finish and execution. Cartwright's talents were just then beginning to be known, and his well deserved fame and success have resulted from the great dexterity, skill and neatness with which he has performed his operations. Thomas Bell, the accomplished writer and lecturer on the anatomy, structure and diseases of the teeth, at Guy's Hospital, was then known and distinguished as a scholar and man of science; and as such he was recognised and received in the highest literary and scientific circles in London. Mr. John T. Edmonds, a young gentleman of considerable professional promise, who became my successor, and to whom I confided my practice, has well sustained himself, and secured a respectable degree of eminence, as a man of tact and cleverness in his profession.

† Dentist to the household of the king of France. His works you have in the library of this Institution—a great and a good man. In a letter which I received from the Chevalier Le Maire, announcing his death, that gentleman bestows a very handsome eulogy on the talents and virtues of Maury—alike creditable to the memory of my worthy preceptor, and to the heart and feelings of his distinguished cotemporary in professional life—the celebrated writer.

And gained the patronage, meanwhile,
Of Baillie, Cooper and Carlisle.†
A brighter hope of wealth and fame
O'er mortal prospects never came,
Than fired my zeal on every hand
From worth the highest in the land.
But there no longer could I stay,
For home and friends were far away:
And bright'ning o'er the broad deep sea
Lay the fair land of Liberty.
There, father, mother, called me home,
Brothers and sisters bade me come;
And they were dearer all the while,
Than wealth or fame in Britain's isle.
My mind, in sooth, was ill at ease,
Ambition could no longer please—
And I resolved to fall or stand
Within my own—my father-land.

And now, though many years have fled,
Numbering my kindred with the dead—
Altho' of every good possessed,
And with great blessings I've been blessed—
Still would I here in justice own,
The high respect in England shown
For cleverness in art attained,
And excellence in science gained,
By men of honored name and birth
Of noblest rank and highest worth.

In proof of this, in our own time,
Numbers from this—their native clime,
Have found their talents there employed,
And England's bounty long enjoyed.

No higher favors e'er were won
By Albion's own most cherished son,

† Dr. Baillie, Sir Astley Cooper, and Sir Anthony Carlisle—physician and Surgeons to His Majesty, George the Fourth.

Than Alston—Newton—realized,
For art here lightly paid and prized:
And louder plaudits there could ne'er
Gladden the heart, or greet the ear,
Nor brighter honors be possessed
Than were awarded to our West.

To me, a stranger in that land,
He freely gave a friendly hand;
And showed me what that hand had done—
What he in childhood had begun—
On what his pencil first he tried,
At his loved father's "Ingle-side;*
Where love and zeal and art combine,
And true affections brightly shine:
For in that little group appear
The forms to memory ever dear—
And when the buds of genius burst,
Of all earth's objects, they were first.
In this rude essay, artists find
The germs of his exalted mind;

*All the pleasure I derived from witnessing the choicest productions of art in some of the most highly celebrated galleries of Europe, would not compare with what I felt, when the venerable West, turning from me, for a moment, re-appeared with the little pannel in his hand, on which, in his childhood, he had attempted to draw the portraits of his parents, brothers and sisters. The softened tone, the kind and affectionate manner in which he spoke of these relatives, and at the same time recurred to the feelings which had prompted the childish effort, sent a thrill of mingled pleasure and melancholy through my whole frame, which I do not remember ever to have experienced either before or since.

During his life he was most graciously received at court, and esteemed as a friend and companion by the highest in the realm. His industry was rewarded with unbounded patronage and success—his talents, professional attainments and high moral character, acknowledged, by his receiving the royal distinction of painter to his majesty—and also by having conferred upon him the presidency of the Royal Academy, which he held for many years. In death, the further distinction was shown him, of laying his body in state at Somerset House—from which I saw the large procession move, that paid the final tribute of respect to his mortal remains—a testimonial of his superior talents, exalted worth, and manly virtues. The pall was borne by men of the highest rank—noblemen, ambassadors, and distinguished academicians, and he was interred with the great in St. Paul's cathedral—the last mark of favor a generous nation could bestow.

For there in lines, unfinished still,
The noble boy evinced his skill.

'Twas there I saw, 'mongst wise and good,
What here is rarely understood—
That greatness never is allied
To scorn—contempt—to pomp or pride;
But gentle words and manners bland
Are marks of greatness in that land;
And condescension, frank and free,
Distinguish great from—*great would be.*

For friendly feeling oft expressed
From many a true and manly breast;
For eulogies on every hand
On my beloved—my native land—
For generous acts of every kind
That go to win an ardent mind,
And signalize the brave and free,
Still England's debtor I must be,
And still must love and cherish those
Who grace old England's laurelled Rose.

By frankest courtesies received,
Which home-sick longings oft relieved—
By pleasure which the traveller finds
In intercourse with lofty minds—
By joys unknown to "ball or bowl,"
"The feast of reason—flow of soul"—
By all that friendship can impart,
Is England hallowed to my heart!
Still there are names among the rest
Of friends the noblest and the best;
Where deepest gratitude is due—
Thrice dearest Hare and Montagu.*

* Thomas Hare, Esq. F. L. S. and Fellow of the Royal College of Surgeons, London.

Basil Montagu, son of the late earl of Sandwich—a name more distinguished for benevolence and philanthropy, displayed both in public and private life—cannot be

Though friends like these made England dear—
America, my home was here;
Where freedom's sons are doubly blest
In our own England of the WEST.

But when I reached my native shore,
Resolved to quit my home no more;
And sought Manhattan's peopled strand—
The great emporium of the land—
Where hearts are warm, and homes are free,
I found that both were closed to me;
For lo! the dentists of that day
Received from patrons "work and pay;"
And when the interview was o'er,
Acquaintance ended at the door;
And rarely could the dentist meet
A recognition in the street,
Excepting from a noble few
To whom my warmest thanks are due.*

found in England's annals. I here transcribe a letter received from him a short time before I left England, to show the warmth of heart and amiable condescension that find an abiding place in the breast of those who hold the highest stations—belong to families of the first rank and wealth—and who constitute the true nobility of England.

*August* 19, 1821.

"MY DEAR SIR,

I, like you, began life, torn from my home, and as I used to say, 'prowling for human prey, without one human sympathy;' but with many heart rendings—many longing, lingering looks towards my country, I persevered. You have attained the object for which you came to England, and you are about to relinquish it. I hope you are acting wisely; money is but one source of happiness—it is the baggage of virtue. You, with your talents, will be able to command it any where. When you quit our island, take one small pebble from the shore; remember our fireside, and remember that, as a father, I offered to consult with you before you finally determined upon a measure which must have considerable influence upon your future prospects. God bless you wherever you go. I beg to know when you can see me—can you appropriate Sunday morning next to me.

Your affectionate,

B. MONTAGU."

* The feeling of dissappointment which I experienced at the condition of things in New York, from the prejudices that seemed to exist, is, perhaps, as fully expressed in a few lines which I wrote, after having spent sometime there in utter loneliness,

That which now makes my bosom swell
With joy—whereon I love to dwell—
Is the advancement that is made
In what was once below a trade;
For, with exceptions very few,
The art, when I began, was new.
Yet, here, the noble Hayden stood;
In Boston—Randall—Flagg—Greenwood;
In Philadelphia, there one met
With Hudson, Koecker and Gardette;
And in New York one scarce could fail
To hear the name of Wooffendale—
And Greenwood too—a worthy man,
And with them Parkhurst and Gaetan.
These are the names which first I heard,
And being first—are still revered.
But many a keen and bitter thrust,
Springing from hatred and distrust—

as I can express it. I had received a letter from England, urging my return, and holding out prospects of higher favors than I had already received. I here transcribe them, for the purpose of inciting you to persevere under trying and discouraging appearances. My introductory letters, which were most cordially written, brought but one uniform result, and that was pain and mortification for having delivered them; and I was kindly advised to go and settle in Albany, as I could do nothing in New York.

The welcome news has come to hand,
And soon the sails will be unfurled
To waft me from my native land—
To me the dearest in the world.

I love my country as my home,
Its laws, its beauties I revere,
Although from it I'm forced to roam
To seek support denied me here.

I fondly hoped as I pursued
My studies far from every friend—
That in returning, they'd be viewed
As something worthy to commend.

But disappointment's been my lot,
And those who promised have deceived—
Ah! would their names could be forgot!
Ah! would to heaven I'd ne'er believed!

But country—home and friends—farewell!
My gladdened soul to England bends—
The place where justice seems to dwell—
That is my home—there are my friends.

Was aimed at men, who in those days
Aspired our infant art to raise ;
Which now, though lightly prized by some,
A valued science has become ;
And slight and disrespect now find
A place but with the shallow mind ;
Good sense and justice ever will
Attest the worth of dental skill.
And if to skill you shall unite
The sterling virtues, that invite
Respect for worth, and truth maintain,
Distinguished honor you may gain.

With pride my bosom also burns
When memory to the past returns,
A few, by generous impulse led,
With Horace Hayden at their head—
(An honored name—beloved—revered—
To truth and virtue long endeared)—
Plans for a Dental Journal laid,
Laws for our Institution made ;
Whose objects are, to cultivate,
In every city, town and state,
Fraternal feelings 'mongst the whole
Who with us shall their names enrol ;
With ardent wish and strong desire
To raise our art and practice higher
In usefulness, as well as grade,
Until with others equal made ;
And thus united, heart and hand,
To elevate, throughout the land,
The standard of our common aim,
And add new honors to its name.
And wheresoe'er we chance to find
Imposture, with its grovelling mind,
That, with the dregs of meanness, shares
In "diamond paste" and "patent" wares,
In "adamant" or "bone cement"—
Or names mis-spelt—with base intent

Of colleagues worthy of such deed—
To suit their *honest* end or need;
Howe'er its "springs" or "tubes" are curled,
We "lash it naked through the world."

From the decided zeal then shown,
This stately edifice has grown;*
And when some future age shall ask,
Who undertook the noble task—
A Dental College to endow,
Like that which greets our vision now—
Fame shall, with accents loud, respond,
A Hayden—Harris—Handy—Bond.

To you, my friends of youthful age,
Ere in your duties you engage,
I would address a word or two—
Touching the life you should pursue.
Although a stranger I appear,
Still do I hold your welfare dear.
In practice, then, you'll sometimes find
False feeling in the public mind;
And oft a narrow view you'll strike,
Esteeming dentists all alike—
Which values gold of purest name
And paste of quicksilver—the same;
Doating on "adamant cement"—
To which some "*doctors*" give assent,
Who oft fall dupes to knaves—not fools—
Apt pupils of Crawcourian schools.†

* The Baltimore College of Dental Surgery.

† The Crawcours were the first to introduce into the city of New York, the filling of teeth with an amalgam of mercury and silver, under the imposing name of "Royal Mineral Succedaneum." For their impositions and dishonesty, they were compelled to leave the country; but they left behind them many who have followed in their track, and become adepts in their principles and practice. It is well known, that they who recommend and use mercury and silver as the best ingredient for stopping all decayed teeth, are totally unacquainted with the nice and difficult

More arrant knaves are rarely found,
Than some who use this vile compound;
Their hearts have blacker spots beneath,
Than "succedaneum" makes in teeth—
Spots hid behind a smirking face,
Fit emblems of their dark disgrace.

'Tis thus the charlatan obtains
Success from others' lack of brains;
Since all discrimination fails,
Where Folly holds th' unequal scales;
In medicine, the highest skill
Stands on a par with—"some one's" pill—
The Dental Art, which Hudson graced,*
Worth so much putty—so much paste—

process of stopping them with gold. I never saw a good operation of any kind performed by one of this class, with which the country abounds; some of whom unblushingly say that their amalgam is superior to gold. Of all such men, I fearlessly declare, that they are void of truth and common honesty. One of these worthies, on being asked if paste was as good as gold, replied—"I consider it so much better, that I have taken out a bushel of the gold plugs of other dentists, and put in paste instead ! ! !"

There would be as much propriety, and greater justice—if such a man as Monroe Edwards was "taken out" of prison and let loose upon the world, and the above fellow "put in instead"—for the one was only a robber of money—but the other was not only that, but a maltreater of teeth, and a reckless underminer of human health. Comparatively, I consider there is quite as great a difference between the two men, as between the two materials—rating Edwards with the pure metal. In Dr. Baker's acknowledgment, (he having advocated its use,) before the American Society of Dental Surgeons, will be found its true character. He said, *"it is a bad filling"—"it is the worst kind of filling"—"it is a nasty filling"—"it is the worst thing in the world to fill teeth with, except as a filling for the mere shell of a tooth that will bear nothing else !"* Such admissions, from such men as Dr. Baker, should make the face of every honest man blush to own he uses it.

* In no part of the world has the dental profession attained to greater excellence than in this country; it received its character from a few individuals, of whom Dr. Hudson of Philadelphia may be ranked at the head. From their successful management of the teeth, a name and credit have been imparted to the profession, which have not only been highly appreciated and rewarded in this country, but also in Europe; as instanced in Mr. Brewster's having had conferred upon him an order of knighthood, for his professional services by the emperor of Russia—and in our esteemed Maynard, of Washington City, having received a gem of great value, from the same distinguished source, for services rendered to the emperor and his

Niagara—with its thund'ring roar—
A famous mill-seat—nothing more.

Yet there are some, whose better sense
Remains the honest man's defence;
These are the artist's cherished friends,
On whom his sure success depends.
Then wheresoe'er your race be run,
Maintain the honors you have won.
Though art may flatter—scorn deride—
Let truth and virtue be your guide;
Integrity—your highest aim—
On this firm basis rest your fame.
Let truth to all your acts extend,
And gentleness with firmness blend;
Each proving still, as best he can,
God's noblest work—an honest man.

family. At the present time, George W. Parmly is receiving patronage and marked favor, at the Hague, from the royal family of Holland, upon whom he is in attendance.

Of the regular profession—those who first took an interest in my practice, and for whose uniform respect and kindness I have always felt grateful—I here beg leave to mention the names of Drs. Wright Post, Alexander H. Stevens, and Valentine Mott. To their high appreciation of the value of good dental operations, I attribute, in a good degree, the beginning of my success in New York, and the change that was wrought in the minds of the citizens, in relation to the estimation in which well instructed dentists and their practice should be regarded.

